# Prevalence of Neonatal Sepsis in a Tertiary Hospital in Jos: A Five-Year Retrospective Study

**DOI:** 10.7759/cureus.84416

**Published:** 2025-05-19

**Authors:** Marcia M Ihekaike, Aderonke Uhunmwangho-Courage, Diamond C Izugbara, Maryam Shehu, Beth K Thielen, Anne M White, Tina M Slusher

**Affiliations:** 1 Pediatrics, Bingham University, Jos, NGA; 2 Pediatrics, University of Minnesota School of Medicine, Minneapolis, USA

**Keywords:** blood culture, morbidity, mortality, neonatal sepsis, neonates

## Abstract

Introduction

Neonatal sepsis is a systemic illness that occurs in newborns. It is a leading cause of neonatal morbidity and mortality globally. The aim of this study is to determine the burden of neonatal sepsis and assess the challenges of diagnosis among neonates hospitalized at the newborn unit of a tertiary care hospital in northern Nigeria.

Methods

Members of the study team extracted clinical and laboratory data from the medical records for all babies admitted to the hospital with neonatal sepsis between January 2015 and December 2019 using a standardized case report form.

Results

A total of 227 (39.5%) were found to have had suspected neonatal sepsis based on clinical presentation. Of these, 134 (59.0%) were male, 59 (26.0%) were preterm, and 93 (41.0%) were hospitalized within the first 24 hours of life. The majority were outborn, 148 (65.2%), and 147 (64.8%) had early-onset neonatal sepsis. Only 10 (4.4%) of these babies had blood cultures done; of these, only one was positive for *Staphylococcus aureus*. The duration of admission was > 7 days in 45.4% of the babies; the parents of 11.5% of the babies signed against medical advice and discharged, and 13.7% died.

Conclusion

The prevalence of suspected neonatal sepsis in our hospital is high, but the rate of blood culture diagnosis is unacceptably low. There is an urgent need for improved laboratory support, including the routine availability of blood cultures and other markers of neonatal sepsis.

## Introduction

Neonatal sepsis is a systemic illness that occurs in newborns, causing significant morbidity and mortality globally [1]. It has been defined as a systemic inflammatory response syndrome in the presence of or as a result of suspected or proven infection with or without accompanying bacteremia, as documented by a positive blood culture within the first 28 days of life [2, 3].

Neonatal sepsis has serious consequences for the well-being of newborns, yet estimates of the global burden and trends in neonatal sepsis are limited, and available studies vary greatly. There were 6.31 million reported instances of neonatal sepsis worldwide, with 230,000 deaths in 2019 [4]. From 1990 to 2019, there was an increase in the incidence of newborn sepsis and a decrease in deaths, with Sub-Saharan Africa (SSA) and Asia bearing the greatest absolute burden [4]. Neonatal sepsis is responsible for an estimated 26% of under-five fatalities worldwide, with SSA having the highest mortality rates [5]. In Nigeria, there is a significant prevalence of newborn sepsis, with neonatal blood culture-positive rates ranging from 22.4% to 37.6% reported in various studies [6-10].

Neonatal sepsis is a serious but neglected public health issue, particularly in low- and middle-income countries (LMICs) in SSA and Southeast Asia. As a result, despite a declining trend in worldwide neonatal death over the previous two decades, the rate of reduction in sepsis-specific mortality in these locations has been substantially slower than that of other causes, such as premature birth or intrapartum problems [11].

One challenge in the diagnosis of neonatal sepsis is the tendency to present with non-specific signs and symptoms. Fever or hypothermia, respiratory distress including cyanosis and apnea, feeding difficulties, lethargy or irritability, hypotonia, seizures, bulging fontanel, poor perfusion, bleeding problems, abdominal distention, hepatomegaly, and unexplained jaundice are some of the presenting signs and symptoms [12].

A high level of suspicion is, therefore, required to make a diagnosis of sepsis in the newborn. Because it is estimated that early detection and treatment of cases can avoid up to 84% of neonatal sepsis-related mortality [13], timely and accurate diagnosis are critical if we are to reduce mortality from sepsis.

As a first step to addressing neonatal sepsis at a tertiary hospital in Jos, northern Nigeria, we performed a retrospective review of cases over the five years to determine the burden of neonatal sepsis as well as to identify the challenges in its diagnosis and treatment among neonates admitted into the Special Care Baby Unit (SCBU). We hypothesized that identifying the challenges in diagnosis and treatment would serve as a tool for improving care, identifying and preventing modifiable risk factors, preventing complications, and advocating for improved health care funding. Therefore, the primary objective of this study is to determine the burden of neonatal sepsis in the newborn unit of our hospital. Our secondary objective is to identify the challenges in the diagnosis and treatment of neonatal sepsis among neonates admitted into the SCBU of the tertiary hospital.

## Materials and methods

Study design

This retrospective case review was carried out on all newborns admitted from January 2015 to December 2019 into the inborn SCBU of Bingham University Teaching Hospital, a faith-based private tertiary hospital located in Jos, Plateau State, Nigeria. Patients were included if hospital records listed a diagnosis of “neonatal sepsis” on their discharge records. Early-onset sepsis was defined as the development of symptoms in the first 72 hours of life [14, 15]. "Inborn" is defined as newborns that are born at the hospital and never discharged; "outborn" is defined as newborns born at another health care facility, in the community, or born at the hospital but discharged before readmission into the SCBU. The case record files of these babies were collected with the aid of a standardized case report template with data entered into Microsoft Excel (Microsoft Corp., Redmond, WA). Data elements included age at presentation, gender, gestational age, birth weight, blood, urine, and cerebrospinal fluid culture, full blood count, malaria parasite test results, and duration and outcome of admission.

Ethical approval

Ethical clearance for the study was obtained from the Health Research Ethics Committee of Bingham University Teaching Hospital with reference number NHREC/21/05/2005/01077.

Inclusion criteria

All neonates admitted during the study period with a clinical diagnosis of suspected or confirmed neonatal sepsis were included. Suspected sepsis was defined using the World Health Organization (WHO) guideline [16].

Exclusion criteria

Neonates admitted during the study period whose medical records were missing or incomplete were excluded from the study.

Data analysis

Data analysis was performed using IBM SPSS Statistics software, version 27 (IBM Corp., Armonk, NY), to calculate descriptive summary statistics. Multivariate logistic regression was used to determine predictors of mortality. Odds ratio situated within a 95% confidence interval (CI) was used to estimate effect size for multivariate logistic regression. The level of significance P was set at < 0.05.

## Results

During the five years under review, a total of 1,657 babies (792 inborn and 865 outborn) were admitted into the SCBU. Of these, the records of 575 (34.7%) babies were found for review. Of these 575 babies, 227 (39.5%) had a diagnosis of suspected or proven neonatal sepsis (Table 1).

**Table 1:** Characteristics of newborns admitted with NNS during the study period NNS: neonatal sepsis; SAMA: signing against medical advice

Characteristics	Number (%) N = 227
Gender	
Male	134 (59.0)
Female	92 (40.5)
Not documented	1 (0.5)
Timing of Presentation	
Early onset (Birth – 72 hours)	n = 147 (64.8)
< 24 hours	93 (41.0)
24 – 72 hours	54 (23.8)
Late Onset (> 72 hours)	n = 79 (34.8)
4 – 7 days	44 (19.4)
> 7 days	35 (15.4)
Not documented	1 (0.4)
Gestational Age	
Term	131 (57.7)
Preterm	59 (26.0)
Not documented	37 (16.3)
Birth Weight	
< 2,500g	69 (30.4)
≥ 2,500g	100 (44.1)
Unknown	58 (25.5)
Place of birth	
Inborn	79 (34.8)
Outborn	148 (65.2)
Duration of Admission	
< 1 day	1 (0.4)
1-3 days	46 (20.3)
4-7 days	74 (32.6)
8 – 14 days	82 (36.1)
15 -21 days	12 (5.3)
> 21 days	9 (4.0)
Not documented	3 (1.3)
Outcome	
Discharged home	169 (74.4)
Died	31 (13.7)
SAMA	26 (11.5)
Referred	1 (0.4)

Only 10 (4.4%) of the babies with suspected sepsis had blood cultures done, of which one was positive (Table 2). *Staphylococcus aureus* was cultured, and antimicrobial testing was sensitive to only erythromycin. The remaining blood and other body fluid cultures yielded no growth. The mean absolute neutrophil count was 7549 cells/µL with a median of 6664 cells/µL.

**Table 2:** Laboratory parameters of babies admitted with NNS in SCBU NNS: neonatal sepsis; CSF: cerebrospinal fluid; TWBC: total white blood cell; SCBU: special care baby unit

Variable	NNS Frequency (%)
Blood Culture	
Done	10 (4.4)
Not done	217 (95.6)
Blood Culture Result	
Positive	1 (10.0)
Negative	9 (90.0)
CSF Culture	
Done	9 (4.0)
Not done	218 (96.0)
Urine Culture	
Done	1 (0.4)
Not done	226 (99.6)
Malaria Parasite Test	
Done	99 (43.6)
Not done	128 (56.4)
Malaria Parasite Result	n = 99
Positive	21 (21.2)
First week of life	16/21
> 1 week	5/21
Negative	78 (78.8)
Full Blood Count	
Done	205 (90.3)
Not done	22 (9.7)
TWBC (mm^3^)	
< 9,000	53 (25.9)
9000 – 30,000	143 (69.7)
> 30,000	9 (4.4)
Absolute Neutrophil Count (cells/µL)	
< 1500	199 (97.5)
1500 - 41000	5 (2.5)

Some babies with neonatal sepsis had some comorbid conditions for which they received treatment along with treatment for sepsis (Table 3).

**Table 3:** Comorbidities among babies with NNS NNS: neonatal sepsis; *Multiple responses, percentages do not add up

Diagnosis	Frequency (%)
Neonatal jaundice	75 (33.0)
Asphyxia	29 (12.8)
Prematurity	59 (26.0)
Malaria	21 (9.3)
Seizures	5 (2.2)
Hypoglycemia	34 (15.0)

Variables such as age, sex, gestational age, birth weight, place of birth, presence of seizures, jaundice, asphyxia, and hypoglycemia were entered into the logistic regression model to determine predictors of mortality. Only birth weight was found to have a significant relationship (Table 4).

**Table 4:** Predictors of mortality among babies with NNS NNS: neonatal sepsis; df: degree of freedom; OR: odds ratio; CI: confidence interval

Variable	df	OR	95% CI	p-value
Age	1		0.000	0.993
Sex	1	0.000	0.814 – 4.436	0.332
Birth weight	1	7.545	1.547 – 36.792	0.028
Gestational age	1	0.000	0.000	0.795

## Discussion

This study found a 39.5% prevalence of suspected or confirmed neonatal sepsis among hospitalized neonates, consistent with reports from Ethiopia and SSA, which show prevalence rates between 25.2% and 39.5% [7-10, 17, 18]. These findings highlight the persistent burden of neonatal sepsis in low-resource settings.

Early-onset sepsis (EOS) accounted for 64.8% of cases, emphasizing the role of perinatal risk factors, including poor maternal health and limited access to skilled birth care [19]. Similar proportions were reported in previous studies in Nigeria and other LMICs [10,20], underscoring the need for improved maternal and perinatal care.

A major limitation was the low rate of blood culture testing, performed in only 4.4% of cases, largely due to financial constraints, lack of insurance, and limited laboratory infrastructure. This reflects a broader trend in LMICs, where culture-negative sepsis predominates [21]. Blood cultures are essential for accurate diagnosis, determining sensitivities, and treatment planning [22, 23], yet access remains limited.

Among the few blood cultures done, *Staphylococcus aureus *was the sole isolate, sensitive only to erythromycin, a finding that may reflect testing limitations or prior antibiotic exposure. The low positivity rate may also result from inadequate blood volumes [24], prior antibiotic use [25], or non-bacterial infections such as malaria, which was detected in 21% of those tested. Other undetected pathogens (e.g., anaerobes, viruses, fungi) for which we currently lack capacity for testing may also contribute. 

The vast majority of babies managed for neonatal sepsis in this study were diagnosed based on clinical signs and symptoms in conjunction with full blood count (FBC) results, a method that, while commonly employed in our setting, lacks the accuracy and specificity of microbiological testing [26]. Additionally, we were unable to perform C-reactive protein (CRP) and procalcitonin [27], which could have been useful tools in determining which neonates were likely to be septic, even in the absence of blood cultures. The reliance on clinical diagnosis, although often necessary in settings with limited laboratory infrastructure, unavailable blood culture bottles, and the inability of families to pay for blood cultures, puts our neonates at higher risk of significant morbidity and mortality than those neonates born in high-income countries. For instance, in 2024, the costs for a CRP, procalcitonin, and blood culture were approximately $6.67, $10, and $10, respectively, in Jos, as compared to a malaria test, which was $0.67.

Birth weight has consistently been identified as a significant predictor of mortality in neonates with sepsis. In the present study, neonates with lower birth weights exhibited a markedly higher risk of mortality compared to those with higher birth weights. This finding aligns with earlier research demonstrating that neonates who succumbed to sepsis were more likely to be of significantly lower birth weight and more frequently categorized as extremely preterm than their surviving counterparts [28, 29].

Low birth weight is a proxy for multiple physiological vulnerabilities, including underdeveloped organ systems, impaired thermoregulation, and immature immune responses, all of which predispose neonates to rapid clinical deterioration in the face of systemic infections. Moreover, low birth weight often coexists with other risk factors such as prolonged hospitalization, invasive procedures, and the need for respiratory support, which further compound the risk of sepsis-related complications and mortality.

One notable limitation of this study is the substantial number of missing patient folders, which restricted the dataset and may have influenced the accuracy of the estimated prevalence and outcomes of neonatal sepsis in our institution. The adoption of an electronic medical records (EMR) system could help mitigate this issue by reducing the loss of patient data and improving accessibility for future research endeavors.

Our findings highlight significant gaps in healthcare funding and policy. We recommend improving neonatal sepsis management by increasing funding and strategic investments in healthcare infrastructure and routine availability of blood cultures and biomarkers such as CRP and procalcitonin. Additionally, the adoption of EMRs is recommended to reduce data loss, enhance clinical documentation, and facilitate research. Furthermore, expanding health insurance coverage to include essential neonatal services would reduce financial burdens on families in low-income settings, enhancing access to life-saving care and promoting equitable healthcare for newborns.

## Conclusions

Neonatal sepsis remains highly prevalent in our hospital, yet blood culture confirmation is rarely performed. Strengthening laboratory capacity, expanding access to diagnostics through improved insurance and government support, and developing clearer clinical guidelines for empirical antibiotic use are urgently needed. A prospective study is also recommended to systematically assess maternal and perinatal risk factors, antibiotic use, diagnostic practices, and treatment outcomes. These measures are critical to improving neonatal health outcomes in Nigeria.

## References

[1] Tewabe T, Mohammed S, Tilahun Y (2017). Clinical outcome and risk factors of neonatal sepsis among neonates in Felege Hiwot referral Hospital, Bahir Dar, Amhara Regional State, North West Ethiopia 2016: a retrospective chart review. BMC Res Notes.

[2] Harrison ML, Dickson BF, Sharland M, Williams PC (2024). Beyond early- and late-onset neonatal sepsis definitions: what are the current causes of neonatal sepsis globally? A systematic review and meta-analysis of the evidence. Pediatr Infect Dis J.

[3] Shane AL, Sánchez PJ, Stoll BJ (2017). Neonatal sepsis. Lancet.

[4] Li J, Xiang L, Chen X, Li S, Sun Q, Cheng X, Hua Z (2023). Global, regional, and national burden of neonatal sepsis and other neonatal infections, 1990-2019: findings from the Global Burden of Disease Study 2019. Eur J Pediatr.

[5] Liu L, Oza S, Hogan D (2015). Global, regional, and national causes of child mortality in 2000-13, with projections to inform post-2015 priorities: an updated systematic analysis. Lancet.

[6] Omoregie R, Egbe CA, Dirisu J, Ogefere HO (2013). Microbiology of neonatal septicemia in a tertiary hospital in Benin city, Nigeria. BGM.

[7] Olorukooba AA, Ifusemu WR, Ibrahim MS, Jibril MB, Amadu L, Lawal BB (2020). Prevalence and factors associated with neonatal sepsis in a tertiary hospital, North West. Niger Med J.

[8] Mustapha SS, Zaidu AM, Azaria NT, Aliyu S, Abdulkadir I (2024). Neonatal sepsis-a peek into our findings in Northwest Nigeria: a prospective study. Gaz Egypt Paediatr Assoc.

[9] Uwe NO, Ezenwa BN, Fajolu IB, Oshun P, Chukwuma ST, Ezeaka VC (2022). Antimicrobial susceptibility and neonatal sepsis in a tertiary care facility in Nigeria: a changing trend?. JAC Antimicrob Resist.

[10] Arowosegbe AO, Ojo DA, Dedeke IO, Shittu OB, Akingbade OA (2017). Neonatal sepsis in a Nigerian tertiary hospital: clinical features, clinical outcome, aetiology and antibiotic susceptibility pattern. S Afr J Infect Dis.

[11] Rafi MA, Miah MM, Wadood MA, Hossain MG (2020). Risk factors and etiology of neonatal sepsis after hospital delivery: a case-control study in a tertiary care hospital of Rajshahi, Bangladesh. PLoS One.

[12] Murthy S, Godinho MA, Guddattu V, Lewis LE, Nair NS (2019). Risk factors of neonatal sepsis in India: a systematic review and meta-analysis. PLoS One.

[13] Roble AK, Ayehubizu LM, Olad HM (2022). Neonatal sepsis and associated factors among neonates admitted to neonatal intensive care unit in general hospitals, eastern Ethiopia 2020. Clin Med Insights Pediatr.

[14] Attia Hussein Mahmoud H, Parekh R, Dhandibhotla S (2023). Insight into neonatal sepsis: an overview. Cureus.

[15] Shane AL, Stoll BJ (2014). Neonatal sepsis: progress towards improved outcomes. J Infect.

[16] (2025). Managing possible serious bacterial infection in young infants when referral is not feasible. https://apps.who.int/iris/bitstream/10665/181426/1/9789241509268_eng.pdf...

[17] Mezgebu T, Ossabo G, Zekiwos A, Mohammed H, Demisse Z (2023). Neonatal sepsis and its associated factors among neonates admitted to the neonatal intensive care unit in Wachemo University Comprehensive Specialized Hospital, Southern Ethiopia, 2022. Front Pediatr.

[18] Traoré FB, Sidibé CS, Diallo EH (2024). Prevalence and factors associated with maternal and neonatal sepsis in sub-Saharan Africa: a systematic review and meta-analysis. Front Public Health.

[19] Maisaba JM, Migisha R, Owaraganise A (2024). Maternal factors associated with early-onset neonatal sepsis among caesarean-delivered babies at Mbarara Regional Referral Hospital, Uganda: a case-control study. BMC Pregnancy Childbirth.

[20] Milton R, Gillespie D, Dyer C (2022). Neonatal sepsis and mortality in low-income and middle-income countries from a facility-based birth cohort: an international multisite prospective observational study. Lancet Glob Health.

[21] Thaver D, Zaidi AK (2009). Burden of neonatal infections in developing countries: a review of evidence from community-based studies. Pediatr Infect Dis J.

[22] Camacho-Gonzalez A, Spearman PW, Stoll BJ (2013). Neonatal infectious diseases: evaluation of neonatal sepsis. Pediatr Clin North Am.

[23] Zea-Vera A, Ochoa TJ (2015). Challenges in the diagnosis and management of neonatal sepsis. J Trop Pediatr.

[24] Woodford EC, Dhudasia MB, Puopolo KM, Skerritt LA, Bhavsar M, DeLuca J, Mukhopadhyay S (2021). Neonatal blood culture inoculant volume: feasibility and challenges. Pediatr Res.

[25] Hirosawa T, Sakamoto T, Hanai S, Harada Y, Shimizu T (2023). Effect of prior antibiotic treatment on blood culture in an outpatient department of general internal medicine: a retrospective case-control analysis. Int J Gen Med.

[26] Odabasi IO, Bulbul A (2020). Neonatal sepsis. Sisli Etfal Hastan Tip Bul.

[27] Yang Y, Xie J, Guo F, Longhini F, Gao Z, Huang Y, Qiu H (2016). Combination of C-reactive protein, procalcitonin and sepsis-related organ failure score for the diagnosis of sepsis in critical patients. Ann Intensive Care.

[28] Jovičić M, Milosavljević MN, Folić M, Pavlović R, Janković SM (2023). Predictors of mortality in early neonatal sepsis: a single-center experience. Medicina (Kaunas).

[29] Belachew A, Tewabe T (2020). Neonatal sepsis and its association with birth weight and gestational age among admitted neonates in Ethiopia: systematic review and meta-analysis. BMC Pediatr.

